# Community perceptions of rape and child sexual abuse: a qualitative study in rural Tanzania

**DOI:** 10.1186/1472-698X-14-23

**Published:** 2014-08-18

**Authors:** Muzdalifat Abeid, Projestine Muganyizi, Pia Olsson, Elisabeth Darj, Pia Axemo

**Affiliations:** 1 Department of Women’s and Children’s Health, International Maternal and Child Health (IMCH), Uppsala University, Uppsala SE-75185, Sweden; 2 Department of Obstetrics/Gynecology, Muhimbili University of Health and Allied Sciences (MUHAS), Dar es Salaam, P.O. Box 65117, Tanzania; 3 Department of Public Health and General Practices, Norwegian University of Science and Technology, P.O. Box 8905, Trondheim, Norway

**Keywords:** Child sexual abuse, Community perceptions, Focus group discussions, Rape, Rural, Sexual violence, Tanzania

## Abstract

**Background:**

Rape of women and children is recognized as a health and human rights issue in Tanzania and internationally. Exploration of the prevailing perceptions in rural areas is needed in order to expand the understanding of sexual violence in the diversity of Tanzania’s contexts. The aim of this study therefore was to explore and understand perceptions of rape of women and children at the community level in a rural district in Tanzania with the added objective of exploring those perceptions that may contribute to perpetuating and/or hindering the disclosure of rape incidences.

**Methods:**

A qualitative design was employed using focus group discussions with male and female community members including religious leaders, professionals, and other community members. The discussions centered on causes of rape, survivors of rape, help-seeking and reporting, and gathered suggestions on measures for improvement. Six focus group discussions (four of single gender and two of mixed gender) were conducted. The focus group discussions were recorded, transcribed verbatim, and analyzed using manifest qualitative content analysis.

**Results:**

The participants perceived rape of women and children to be a frequent and hidden phenomenon. A number of factors were singled out as contributing to rape, such as erosion of social norms, globalization, poverty, vulnerability of children, alcohol/drug abuse and poor parental care. Participants perceived the need for educating the community to raise their knowledge of sexual violence and its consequences, and their roles as preventive agents.

**Conclusions:**

In this rural context, social norms reinforce sexual violence against women and children, and hinder them from seeking help from support services. Addressing the identified challenges may promote help-seeking behavior and improve care of survivors of sexual violence, while changes in social and cultural norms are needed for the prevention of sexual violence.

## Background

Violence against women and children has gained international recognition as a grave social and human rights violation during the last few decades. The underlying causes and contributing factors of violence against women and children are deeply entrenched in community traditions, customs and culture [1,2].

Gender-Based Violence (GBV) refers to violence that occurs within the context of women’s and girls’ subordinate status in society characterized by power imbalance in the home and society at large [3]. GBV occurs on a vast scale and takes different forms throughout women’s and children’s lives, ranging from Child Sexual Abuse (CSA), early marriage, female genital mutilation, rape, forced prostitution, and wife beating, to the abuse of elderly women [4]. The term GBV can be used interchangeably with “violence against women”; however, the latter is a more limited concept. This study focuses on rape against women and children, for which we used the term “sexual violence”. Rape was defined as sexual contact that occurs without the woman’s consent, involves the use of force, threat of force, intimidation, or when the woman was of unsound mind due to illness or intoxication and involves sexual penetration of the victim’s vagina, mouth or, rectum [5,6]. We preferred this definition to the legal definition of rape in Tanzania, which does not recognize marital rape.

Globally, it is estimated that between 14% and 25% of adult women have been raped and the prevalence of CSA varies between 2% and 62% [7-12]. In Tanzania, physical or sexual violence by an intimate partner is reported by 44% of ever-married women aged 15-49 years [13]. The same survey showed that 39% of the total sample of ever-married women reported having experienced physical violence, while 20% of the total reported having experienced sexual violence in their lifetime. Nearly 1 in 3 females and approximately 1 in 7 males in Tanzania have experienced sexual violence and almost three-quarters of both females and males have experienced physical violence prior to the age of 18 [14]. The most common type of childhood sexual violence was unwanted touching (16% and 8.7% of females and males, respectively) followed by attempted unwanted intercourse (14.6% and 6.3% of females and males, respectively). Almost 6.9% of girls and 2.9% of boys were physically forced or coerced into sexual intercourse before the age of 18. Over 60% of girls who did not disclose incidences of sexual violence to anyone gave family or community reasons (with the most common reason being fear of abandonment or family separation), while another 26% gave personal reasons. For boys, 58% gave personal reasons (with the most common reason being not thinking it was a problem), while 36% gave family and community reasons (not wanting to embarrass their families, afraid of being beaten, did not think people would believe them) [14]. Only about 1 out of 5 girls and 1 out of 10 boys seek legal or health services after their experience of sexual abuse. Of those, only 1 in 10 girls and 1 in 25 boys who experienced sexual violence received any kind of service [14].

Most Tanzanian communities follow the patriarchal kinship pattern whereby the inheritance and power is vested in the husband’s clan [15-17]. Women lack decision-making power in various matters including how, when and where to have sex. Many ethnic groups are polygamous and condone the practice of multiple sexual partners. Sexual practices such as *“Chagulaga mayu”* which means, “choose one among us”, are not uncommon. *Chagulaga mayu* is practiced among the biggest ethnic group in Tanzania, the ‘Sukuma’ from the Mwanza region, around harvest time where festivities accompanied by traditional dances mark the occasion [17]. Unmarried women who attend the dances are chased around by men until the men choose those with whom they will have intercourse at the end of the ceremony. Sometimes these casual sexual incidences culminate in marriages [15-17]. These traditions and practices are widespread and occur in other regions of Tanzania due to the migration of people, and illustrate some of the pitfalls associated with patriarchy when it comes to gender relations. The patriarchal nature of this tradition of *chagulaga mayu* means that it leaves the women with no choice but to accept the sexual act because, culturally, it is accepted that the women must submit to men’s wishes. Patriarchal structures benefit men more than women, where women are culturally considered to have a subordinate status and minimum influence on decision-making, even in regards to their own health.

Some of the practices that perpetuate gender inequalities and power imbalances include initiation rites through a special kind of training; *jando* (for boys 14-20) and *unyago* (for girls 11-20). This training is intended to prepare them for family and social responsibilities as adults. Some of the ethnic groups in Tanzania who continue to practice such initiation rites include the Zaramo and Makonde [15,17]. In most communities, these processes are centered on circumcision, whereby reputable elderly people are entrusted with these tasks. While girls are prepared for reproductive roles and family responsibilities, boys are prepared to be future leaders, from family to society level. Such initiation rites have taken on different forms depending on the socio-economic and cultural context of the individual community. Among matrilineal groups, where women have some political and economic power, it has been a legitimate social objective to maximize pleasure [17]. Among patrilineal societies, however, the roles of men and women are very much differentiated, and taboos often support the characterization of women as being polluted and likely to pollute. The practice of female circumcision is associated with the curtailment of female pleasure and a mechanism whereby men can control women’s sexual activity [15,16]. The initiation ritual is still a mechanism for defining womanhood in some contemporary societies in Tanzania. However, these are rapidly losing ground. Some elements of these traditions have been abandoned, and the rituals fail to accommodate new social needs. The original messages about responsible parenthood and what it entails to become a sexually active adult have been diluted and are no longer relevant to adolescent change in present-day society. Today, young people are presented with conflicting values and are given no clear guidance on standards of behavior and little information about matters of sexual and reproductive health. The fragmentary information they do acquire comes from their peers and the media. The taboo for mothers to discuss sexuality with their daughters is still upheld [15-17].

In the last few decades in Tanzania, major social change has taken place which has had an impact on the expression of sexuality and its consequences for adolescents and youth. In contemporary society, marriage of young people is delayed because of their increasing engagement in schooling and income generation. Previously, the only legal way of gaining access to female sexuality and fertility was through bride wealth; a marriage system in which the rights to a woman are acquired through the transference of goods from the groom’s to the bride’s kin [18-20]. Today in Tanzania, young girls and women engage in transactional sex and see it as a “normal” part of sexual relationships, motivated by a desire to acquire modern commodities [19-22]. Young women and their sexuality can be used, even by their mothers, for economic benefit [23]. Although girls have negotiating power over certain aspects of sexual relationships such as those with an older man, a *Mshefa or buzi,* they have little control over sexual practices within partnerships, including condom use and violence [18-23]. In actual practice, female sexual behavior allows extensive sexual networking, exacerbating their vulnerability to HIV infection and thus, because of biological reasons, they are more prone to infection [18-25].

The Government of Tanzania is making efforts to address GBV through legislation, policies, and strategies in all social spheres of life. The *National Policy Guidelines for the Health Sector Prevention of and Response to Gender-based Violence 2011,* outlines the roles and responsibilities of the Ministry of Health and Social Welfare and other stakeholders in the planning and implementation of comprehensive GBV services; while the *National Management Guidelines for the Health Sector Response to and Prevention of Gender-based Violence 2011,* provides a framework for standardized medical management of GBV cases and aims to strengthen referral linkages between the community and service providers. These laws and policies provide an intrinsic link between GBV and health rights in relation to HIV/AIDS, and reproductive and child health morbidities and mortalities. However, while the principle of gender equality is enshrined in the Constitution of the United Republic of Tanzania, legal protections against GBV are limited. The Tanzanian Sexual Offence Special Provision Act (SOSPA) specifies that; “A man who has sexual intercourse with a female below 18 years of age, with or without her consent, has committed rape unless she is his wife and above 15 years of age and not separated from him” [26]. This law states that the punishment for the convicted rapist is a minimum of 30 years of imprisonment [26]. However, the Marriage Act does not recognize marital rape and further allows for child marriage at 15 years of age with parental consent [27], thus ignoring a substantial proportion of women in Tanzania who express concern about marital rape.

Sexual violence is highly gendered, and associated with numerous health consequences. The third Millennium Development Goal (MDG) is to “promote gender equality and empower women”, and many governments, including Tanzania’s, are increasingly focused on addressing issues related to gender [28,29]. However, the understanding of how gender intersects with health and ill-health is often limited [30] and gender theory could help further this understanding. The relational theory of gender by Connell [31,32] depicts gender as being multidimensional and encompasses, at the same time, economic, power, emotional and symbolic dimensions that operate simultaneously at intrapersonal, interpersonal, and institutional and society-wide levels.

Previously, community perceptions of sexual violence, disclosure of events and support to survivors have been investigated in urban Tanzanian settings [33-36]. Variations in awareness of services and their availability, non-existent formal referral networks that integrate across services and numerous other barriers often prevent women from seeking help [28,33-36]. To widen and deepen the understanding of sexual violence in its diverse contexts and to enable policy development and interventions relevant to the entire country, the exploration of perceptions prevailing in rural areas is needed. Understanding the strengths and gaps in existing support services, as well as community needs and potential barriers to care in rural settings, is of utmost importance in order to increase the availability and utilization of GBV services. The aim of this study was to explore and understand perceptions of rape of women and children at the community level in a rural district in Tanzania. The objectives were to explore the perceptions that may contribute to perpetuating and/or hindering the disclosure of rape events in the study context.

## Methods

### Study design

An inductive qualitative design employing focus group discussions (FGDs) [37] and qualitative content analysis (QCA) [38] was chosen for its potential to enable identification and exploration of contextualized community perceptions of rape of women and children. The term FGDs refers to a method of data collection that gathers people of similar backgrounds to discuss a research topic [37].

### Study setting

The study was conducted in Kilombero, a district in the Morogoro region in the Southeastern part of Tanzania. The district has a total population of 416,401 and a literacy rate of 24% [39,40]. The area is predominantly rural with the semi-urban district headquarters, Ifakara, located 420 kilometers south-west of Dar-es-Salaam, Tanzania’s biggest commercial center [39,40]. The major features found in the Kilombero district are the Kilombero River and the Udzungwa Mountains. The river separates the Kilombero district from the Ulanga district while forming the vast Kilombero valley floodplain. Large parts of the valley are flooded during the rainy season, between November and May. In most rural areas, the roads are in very poor condition, so during the rainy season they may be impassable, leaving some areas disconnected from the rest of the district. The economic activities of the Kilombero residents are mostly subsistence farming, fishing, animal husbandry and petty trade, although most people rely on subsistence farming. This district is unique in that it has large sugar cane plantations that employ laborers who are recruited from different parts of the country. During cultivation months, parents move away from their children to these remote sites, which are under intensive cultivation. The cultivation period may last for six months. The residents of Kilombero belong to the indigenous ethnic groups of Ndamba and Pogoro and others who have migrated from the nearby regions, including Hehe and Sukuma. Traditionally, these ethnic groups are organized into patrilineal clans [15,16]. Currently, polygyny formally occurs among the Swahili Muslims only but it is not uncommon for other ethnic groups to have an informal ‘second wife’ (referred to as *kidumu,* literally, ‘small wife’). The families are extended and fathers live with their partners and children. The common household strategy is for the husband to leave the wife/partner to do the agricultural work while he goes fishing. The unmarried young mothers, together with their children, continue to live and depend on their parents. The cultural practices among the Ndamba and Pogoro are not documented; what is evident in the field is that they have adopted the cultural practices inherited from the migrated ethnic groups, such as the *chagulaga mayu, unyago* and *jando*.

### Study sample and data collection

#### Participants

In total, 54 community members, aged 18 to 58 years, took part in six FGDs. A purposive sampling technique was used to obtain the informants for the FGDs. To allow for maximum variation of perceptions within a sample size suitable for FGDs, we recruited participants from different ages and social groups. The recruitment was performed by the first author (MA) in collaboration with the village and ward leaders in the district headquarters. The participants included were professionals (doctors, nurses, lawyers, teachers, police); religious leaders (Catholics, Muslims, Pentecostals, Seventh-Day Adventists, Moravians); and other community members (farmers, housewives, students, village/ward executive officers). An overview of the FGDs is given in Table 1. All the FGDs were homogenous with respect to gender except for FGDs 3 and 4. The rationale for using groups of mixed gender was that previous research had evidence of obtaining open and rich discussions in mixed FGDs on sexual violence in Tanzania [33-36]. Because we wanted to have the discussions with the participants as they normally interact in their daily lives, we recruited 2 groups of religious leaders and separated them by gender. Sexual violence survivors were not actively recruited for these discussions; however, given the prevalence of violence in the country, it is likely that individual survivors, as well as friends and family members of survivors, did participate in the groups.

**Table 1:** Characteristics of FGD groups and participants

**Group**	**Participants**	**Age (range in years)**	**Female/male**
1	Religious leaders	40-55	0/8
2	Religious leaders	38-50	6/0
3	Professionals	34-57	6/5
4	Other community members	29-58	5/5
5	Young men	19-23	0/9
6	Young women	20-23	10/0

#### Focus group discussions

Data were collected in six FGDs during three months in 2012. The ability of FGDs to utilize group interactions for exploring people’s perceptions at societal level makes this method of data collection suitable for investigating community perceptions of sexual violence in their cultural context [37,41]. Because the topic was sensitive, FGDs were held in a small rented conference hall that allowed privacy in the discussions without interruptions. They were all conducted in Swahili and led by an experienced FGD moderator and one of the co-authors (PM). The first author (MA) assisted the moderator, observed the non-verbal activities and took field notes. The FGDs’ point of departure was a local newspaper article [42] depicting rape cases in a rural district outside Kilombero. The title was read aloud: “Anayetuhumiwa kubaka mtoto afariki ghafla” (*The one charged with child rape dies suddenly)*. Two parts of the article were read aloud. The first part was on the statistics showing an increased reporting to the police of rape of children under18 years. The second part told that most rape cases are dealt with at the family level by giving economic compensation to survivors. Thereafter, the question: ‘*How is the situation in this district?*’ was put to the group and the discussion started. A topic guide was used which centered on: (a) causes of rape, (b) survivors of rape, (c) help-seeking and reporting, and (d) what would change the situation. Information from the first FGD guided probing in the subsequent FGDs. After the sixth FGD, the research team felt that no additional information was being given. All FGDs were tape recorded with the participants’ consent and lasted between 90 and 120 minutes. Participants were served soft drinks but no other incentives were provided.

### Analysis

Prior to the analysis the first author transcribed the FGD information using both the tape recordings and written notes. These Swahili transcripts were then translated into English to enable non-Swahili speaking researchers to take part in the analysis. One of the transcripts was back-translated to Swahili by a professional translator to ensure accuracy of the translation. Minor corrections were made. Manifest qualitative content analysis was used to analyze the English transcription [38]. QCA was chosen because it allows systematic organization and analysis of data. The first step in the analysis was to read the transcripts several times to get an overall understanding of the messages in the text. Meaning units were identified and condensed to retain the core meaning. These condensations were further shortened to codes or labels which were sorted into sub-categories and categories with shared commonalities. The process of analysis is depicted in Table 2. Discrepancies that emerged during analysis or interpretation of results necessitated referring back to the transcripts and these were discussed until consensus was reached among authors.

**Table 2:** Example of the analytical process in an excerpt of an interview text

**Meaning Unit**	**Condensed MU**	**Codes**	**Sub-categories**	**Category**
*I see many rape cases, those reported are when a child have already shown signs of being injured or has had big complication that is obvious*	Rape cases are common but only serious cases involving children with revealing complications are reported	Reported if survivor is a child.	Rape is common	Rape is a common and hidden problem
		Reported if there are severe injuries	Reporting based on age and severity	

### Ethical consideration

The study topic of sexual violence is of a sensitive nature. To ensure informed voluntary participation, the study participants received an oral explanation of the objectives and procedures of the study. They were told how confidentiality would be safeguarded and were also fully informed of the potential risks and benefits of their participation [43]. The FGDs were held in privacy. A professional counselor was placed on site to address participants’ emotional needs resulting from their experiences of sexual violence should the need arise. Participants were urged to keep the information discussed within the group confidential. The research team obtained ethical clearance from the Ethical Committee of Muhimbili University of Health and Allied Sciences (MUHAS). Local permission was sought from the Kilombero District Council. The research team strictly adhered to the WHO safety and Ethical Guidelines for Researching Violence Against Women [43,44].

## Results

After the data were analysed, six key categories in the community’s perceptions of the rape of women and children were identified:

1. Rape is a common and hidden problem

2. Abandoning traditional values and modeling Western behavior contributes to rape

3. Suboptimal child care contributes to child rape

4. Survivors of rape are blamed for disclosure of the act

5. Insufficient, costly and corrupt support services are a barrier to help-seeking

6. Collaboration of key stakeholders is needed to improve help-seeking

### Rape is a common and hidden problem

Overall, participants defined rape as sexual intercourse with a child below 18 years of age or with an adult who has not consented to the act. They perceived that boys, girls, men, and women could be raped. However, the majority of young female participants were not aware of what really constitutes rape and who the perpetrators/survivors are. The participants demonstrated recognition of the short- and long-term consequences of rape and cited trauma manifestations such as injuries to the genitalia, including bruises, bleeding or foul-smelling discharges, and acquisition of chronic diseases, such as STIs, HIV/AIDS, fistula and the inability to conceive. The psychosocial impact of rape was perceived to manifest itself as poor performance of children in school, and withdrawal from social interaction. The participants acknowledged that these sexual violent acts are common and condoned by communities. They also perceived that these acts require a response such as reporting the incident to the police or require other complementary services such as health or legal aid. Rape was perceived to be seldom reported due to several barriers that favor the acceptance and non-disclosure of sexual violence.

*In general, there are many cases and I can say those which are reported are few compared to those which are happening around, and the big problem is the environments of rape normally are very private, one cannot rape in an open environment, the environment must favor the thing (rape).* (FGD, Professionals)

Rape by a stranger, especially the rape of a child, was defined as an unacceptable form of violence deserving harsh penalties. But the rape of a child perpetrated by a known person or relative was not normally disclosed to legal authorities as reporting it was seen as putting the family’s honor and reputation at stake.

*So, when the perpetrator comes from another village he will be imprisoned. But if he is a relative, like an uncle, cousin or brother, the community must hide this case.* (FGD, Female religious leaders)

Throughout the discussions, however, participants insisted that the rape of older children in most cases are not reported and are often accepted within the social and cultural norms of their communities.

… *girls of 12-14 years and above most of the time are not reported as rape cases.*

*… they are seen as normal acts. It is the responsibility of the girls (as breadwinners) at that age.* (FGD, Community members)

However, across all groups, a critical distinction that determines help-seeking behavior was made between rape and ‘forced sex’. The most prominent perception was that within marriage, the man who forces his wife to have sex is not considered to have committed rape; hence he has not committed an illegal act or crime. This was based on the assumption that the national law requires that a married woman is ready to fulfill her husband’s sexual desires at all times, even when the act is forceful.

*The law states, if a woman is married it means anything that the husband does should be humbly received even if it involves being raped. If you (as a married man) know the law, you will defend yourself in that you are not raping her but are making love. A wife cannot tell him that he has raped her because the moment you are married it means you consent to it.* (FGD, Community members)

While both men and women overwhelmingly agreed with this distinction, one participant proposed that non-consensual sex within a marital relationship should be considered rape. The view was not agreed upon by other group members who were referring to the Tanzanian legal and social contexts, which do not recognize marital rape.

Here is where another problem arises as it is said; equal rights for both genders, men and women. It’s the husband’s right at that time (when he wants sex) and if the wife is not willing then it means he should not (force her to sex), otherwise it’s rape.

(FGD, Community members)

### Abandoning traditional values and modeling Western behavior contributes to rape

Participants attributed the increase in sexual violence to a number of factors, such as gradual transformations in society that are seeing traditional social norms being replaced by the values and practices of Western society. Poverty, poor parental care, alcohol/drug abuse, and the influence of globalization that has promoted the acquisition of foreign Western cultures, partly through exposure to internet and television, has greatly impacted on social behaviors and relations. Although the majority of the participants criticized some old traditions and considered them to be harmful to society at large, some traditional practices were still acknowledged as being valuable. The ‘*chagulaga mayu*’ with the literal meaning, ‘choose one among us’ was considered to be harmful. Some old traditions, such as ‘*unyago*’ (young women’s initiation) were still embraced. *Unyago*, when conducted by women of strong moral repute, was perceived as conveying family traditions and customs, and contributing to a good future family life. It was lamented that *unyago* has drastically diminished and now is increasingly being replaced by what is now known as ‘kitchen parties’ which are perceived to be an urban practice in which the bride-to be receives teachings on sexuality and how to become a noble wife prior to wedding ceremonies. Unlike in *unyago* ceremonies, these urban teachings (kitchen parties) are conducted publicly and used as platforms for sexual advice and demonstrations. Men, who visit some of these public places where kitchen parties are held, enjoy access to shared information. The participants revealed that this form of initiation is perceived as being immoral because it provides an opportunity for unmarried women to have open discussions about issues that might lead them to engage in sexual activities.

*Talk about traditions* ***,*** *these days said to be changing with time it’s called “A kitchen party”. If you attend these functions, you will see shameful things happen there. When you look carefully at the crowd it is mostly young women, discussing and advising on issues way above their ages and using enhancers like a microphone.* (FGD, Female Religious leaders)

*I never knew where babies came from until when I was thirteen or fourteen years old. But now a child knows everything; how to make love he sees on TV, and there is a lot of TV exposure and this instigates action – they want to try out … But in the past things like that were not common in our society. So they fought ‘Unyago’ (earlier initiation practices) but now something else replaced it”.* (FGD, Professionals)

The role of lifestyle behavior, such as men’s use of alcohol and drugs, having a mistress and women wearing Western dress, were discussed lively in all FGDs. Alcohol was linked to the husband’s use of violence towards his wife, including forced sex and rape. Wives suspecting or knowing that the husband had a mistress would try to avoid having sex with him to prevent getting HIV/AIDs, but such attempts could eventually lead to forced sex or rape. Women’s use of Western dress – wearing tight, transparent and short clothing – was perceived to arouse men’s sexual desire which would be followed by their demand for sex and the likelihood that they would be forceful about it.

*Some girls are wearing miniskirts and go to the well to fetch some water… so it is not uncommon for them to be raped. So girls’ clothes are contributing to this (rape).* (FGD, Young Men)

*This (rape) is caused by the use of drugs and alcohol … Old people say they are drinking beer so as to avoid stress, and youth drink beer and smoke marijuana which pushes them to do strange things, not only rape, even stealing.* (FGD, Male religious leaders)

### Sub-optimal child care contributes to child rape

In all the FGDs, participants expressed deep concerns about poverty and poor parental care that make children vulnerable to several risks including sexual abuse. Some participants reported that parents’ poor economic status might force girls to engage in risky sexual activities in order to solicit financial support from boyfriends or engage in prostitution.

*Poverty is a problem that pulls us into other problems. Humanity is on sale at the moment. … Very often we see that parents themselves send their daughters to go do these things prostitution) so as to bring something home.* (FGD-Female religious leaders)

The participants also claimed that children could become victims of the ‘wealth myth’, meaning that men believe they will become rich if they have sex with a child. The young men in the FGDs perceived that the so-called Freemasons’ Society (believed to be a secretive society of men who have the power to manipulate and control others with their wealth) is the source of the youths’ strange behavior as they instruct the younger generation to do strange things, including rape for economic benefit. Participants across all age groups and of both genders expressed concern that there was poor parental monitoring with regard to girls, especially after they reached puberty when they became more vulnerable to negative peer influences. This issue was more pronounced when parents would leave their children alone while they tended to the cultivation of their farms. Such circumstances were perceived to provoke the girls to solicit financial and emotional support from elderly men. Some parents in vulnerable life situations were depicted as being less cooperative when their children misbehaved at school or when the children were caught by the police due to misconduct.

*Leave my child alone, because I myself am a prostitute, and am earning an income to take her to school.* (FGD, Female religious leaders)

### Survivors of rape are blamed for disclosure of the act

Fear of being blamed for both reporting rape and the stigmatization of women for the rape they experienced was perceived as a powerful hindrance for disclosure of rape in this context. The discussions revealed that women reporting rape were not always believed to be telling the truth and could be blamed for having consented to sex. Wearing short skirts meant inviting sex or rape and these women were to be blamed if they were subjected to unwanted sexual advances or sexual assault. Women’s fear of their partner’s reactions and concerns about the family’s reputation were also barriers to disclosure of rape. Reporting rape and making the act known publicly was perceived to shed shame and dishonor on the family. As rape within marriage was considered to be a private issue and not a crime, it was not likely to be shared with people outside the relationship and was very seldom reported. If the rape victim was unmarried, disclosure would reduce her chances of getting married. Women were perceived to suffer the pain and anguish after rape in silence, fearing the consequences of disclosure. As a Swahili saying goes; ‘*kufa na tai shingoni’* (die with the neck tie).

*When the child gets raped, for the mother to disclose such an incident is not easy, it is a shame on her because it’s like exposing herself naked in front of society. Sometimes the society expresses views that it was not truly rape but the result of negotiations and agreement between the two … also out of fear that young men would not make them their bride. A man cannot pick a sexual violence survivor as fiancée.* (FGD, Male religious leaders)

*We (women) are ashamed to speak up in front of everyone about what has happened as married women. We fear they will call us liars which is a major problem that many cannot bear to face. We’ll look like the one who is breaking up the relationship.* (FGD, Community members)

### Insufficient, costly and corrupt support services are a barrier to help-seeking

Cost, corruption, distance, limited services, and lack of quality care services were seen by participants as barriers to care-seeking behavior and reporting on sexual violence by survivors. The police posts and health facilities are scarce and have to cover a wide geographical area and the informants perceived this to be a major hindrance to obtaining care at an appropriate time. Seeking care was felt to be an additional financial burden as they would need to work extra time in order to meet the expenses of transportation and other services. Informants revealed a sense of frustration with those in the legal sector. The police responded slowly to reported rapes and this was explained as being because rape was a low priority issue for them. They indicated that rape was not treated as an emergency, either by the police or the judiciary. It was perceived that the outcome would be determined by who you know and how much money you have and are willing to provide. They portrayed that reporting rape does more harm than good; often the perpetrator escapes prosecution and the survivor is left without justice and is left only with shame. Participants urged that restraint should be placed on the power of law implementers; they expressed deep disappointment in the police and judiciary sectors and reported that corruption within these departments hindered the implementation of the laws against sexual offenses.

*Let me say one thing - police are corrupt. If you are poor and your son is accused of rape, he will go to jail. But if you are rich you will talk to the head of police and the case is closed.* (FGD, Young men)

The existence of the SOSPA law was expressed by the participants as being a significant symbolic achievement in the effort to strengthen women’s rights and reduce violence against women. However, they perceived that the implementation of the law has been difficult as the law enforcement institutions are under-funded, inaccessible and incompetent. The participants in most FGDs expressed the view that the sexual offence law was also not implemented because the community accepted these acts so as to protect each other as well as the community’s dignity. They perceived that the stringent laws convicting perpetrators to many years of imprisonment is not helpful, and because of that they would prefer to get financial compensation or even make the survivor marry the perpetrator.

*These long punishments behind bars don’t help much. As you have seen, we live in the community. Maybe when you look at the people here you will find that they come from the same family or same tribe. And if they find out that their relative is guilty and will be jailed for 30 years or for life, they cannot accept it, so, as a community, we are used to defending ourselves.* (FGD, Professionals)

### Collaboration of key stakeholders is needed to improve help-seeking

The fear of perpetrator retaliation was mentioned as one of the reasons that sexual abuse survivors do not use formal channels of help-seeking such as the health facility or the police. This was reported to be more pronounced among child survivors, who were threatened by the perpetrators not to disclose an incident otherwise they would be killed. Healthcare was perceived to be reserved for severe physical injuries or when survivors require forensic evidence to pursue legal actions. Survivors who wished to report sexual violence turned to their community leaders primarily for advice and referrals within the local government hierarchy as well as to healthcare facilities and marital reconciliation services. Survivors who were not severely physically wounded were discouraged by community leaders from pursuing prosecution and were instead reported to be encouraged to accept economic compensation to protect the community’s honor. In the case of marital rape, because of shame and stigma, survivors would seek solace from elderly family members, friends or religious leaders.

*Most of the time these kinds of issues (marital rape) involve and end up with elders. They are not taken to the local government leaders or anything of that sort, only to those elderly people with wisdom. If impossible to solve, then maybe they take it to the village chairman. But the chairman himself is not involved, rather it’s the elderly family relatives and they sit together and solve it.* (FGD, Female religious leaders)

Across all the FGDs, the participants pleaded for improvement in preventing and managing cases of rape at the community level. Participants conveyed the view that rural communities lack education and this could be a major barrier to seeking justice. They perceived the need to educate the community across all age and social groups to raise their knowledge on issues pertaining to rape, the health consequences, and the importance of seeking care, and the laws that exist to support the survivors or prosecute the perpetrator.

We know education in Tanzania hasn’t reached all citizens. People have education but it is the most basic primary education. In the interior, villagers don’t even know how to read and are the ones at risk of getting into serious trouble. How do you expect this kind of person to stand in front of the law and fight for their rights?

(FGD, Female religious leaders)

In addition, participants indicated that more training for police and healthcare workers was needed. They also wished for changes in the judicial system that would make prosecution easier for survivors, not for offenders. Nevertheless, the informants underscored the need for key actors, religious leaders and public institutions to collaborate and aggressively condemn such offenses.

*I thought there could be a session which will teach the religious leaders on how to educate society on these (rape) things. Sometimes you might ask why they (religious leaders) fail to advise them (community members). But maybe they have no knowledge. So if the government collaborates with the pastors, it is possible to pass down the information to the members and prohibit such acts.* (FGD, Male religious leaders)

## Discussion

This qualitative study revealed that in this rural Tanzanian community, rape of women and children was perceived to be a frequent and hidden phenomenon, which is on the upsurge and is exacerbated by poverty, alcohol/drug use and a rapidly-changing system of norms. Disclosure and seeking help following rape was perceived to be hindered by stigmatization and structural barriers. To illuminate the dynamics of sexual violence and gender relations in this study context, we will frame the discussion using Connell’s relational theory of gender [32]. We will proceed to discuss the analysis from the four dimensions of gender described; economic, power, symbolic and emotional.

### Economic dimensions

The economic dimension of gender relations was prominent at different levels in the study context. Poverty emerged to be the major constraint in not only accessing care and meeting the expenses of care, but also rendered women and children to be the most common victims of sexual violence. At an individual level, we find that poor economic status made adults and children more vulnerable to prostitution in order to solicit financial and material support, eventually becoming victims of sexual violence. There is a clear connection between sex and economic survival for young women, ultimately rooted in conditions of poverty, disadvantage and lack of opportunity. Our findings are in line with previous research done in other parts of Tanzania that associated poverty with young people’s vulnerability to perpetrators who offer them financial support [19-23,45]. Poor economic status also contributes to the survivors opting for economic compensation from the perpetrator rather than pursuing justice through the legal system. The economic dimension of gender relations was also evident at both societal and institutional levels when the survivors of sexual violence were obliged to incur substantial costs in order to seek and receive care. Corruption, for example, could prevent a woman from accessing justice if the perpetrator had the means to “pay off” the police or local government officials. This finding, which is in line with a study of CSA done in a semi-urban area in Tanzania [35], contributed to the low rate of help-seeking observed among women and children. The present study illustrates how the responses of criminal justice professionals affect not only prosecution, but also influence whether a woman reaches out to other support services as well. Police can serve as an entry point for rape survivors into different support channels. Previous studies have demonstrated that rape survivors who reported to the police were more likely to receive medical care and other referrals [46]. Economic power, as seen in a partner’s alcohol abuse and extramarital affairs, was linked to intimate partner violence, including rape, as demonstrated in other studies in Sub-Saharan Africa [47,48]. A woman’s dependence on a man to support her financially would force her to remain in a violent relationship. The complexity of sexual violence and its association with poverty, the influence of globalization, and alcohol and substance abuse, suggest that single programs are unlikely to produce long-lasting change and that comprehensive approaches are needed. Establishment of a community-coordinated system of care that involves vital social systems such as the police, legal and medical systems, rape crisis centers and the religious community, can have profound implications on a survivor’s recovery and the key focus must be on the prevention of secondary victimization, which includes: victim blaming and inappropriate post-assault behavior or language by medical or legal justice personnel [49]. However, for the system to be successful, it is important to target the perceived barriers by providing education and economic empowerment to the communities. Income-generating schemes like the IMAGE intervention in South Africa, a program that combined microfinance (the provision of loans to poor households) and gender training, has been shown to reduce the level of violence among program participants to half, and highlights the benefit of facilitating economic empowerment of women, indicating that the establishment of gender norms is necessary to ending violence [50].

### Power dimensions

The power dimension of gender relations was reflected profoundly at different levels within the FGDs. At the societal level, non-consensual sex within marriage was not considered to be rape, and the subordinate status of women was reflected when the community members regarded marriage as entailing an obligation of women to be sexually available virtually without limit. In this community we found the ideology of male superiority to be strongly portrayed in the *chagulaga mayu* practice, leading to an increase in sexual violence [10]. Power relations between women and men were seen to be changing as the norm systems change. Community members had started to challenge some of the traditional gender and social norms that are supportive of violence. As part of this transition, the struggle to uphold traditional values was stronger and more obvious as they expected women to maintain a submissive attitude; findings which are consistent with other studies in which gender inequalities are maintained by gender norms which promote men’s superiority over women [2,51]. Men’s control over women can lead men to have an interest in seeking redress from perpetrators. However, it is a great challenge to obtain legal justice in rural areas as they must travel great distances to reach police posts and courts, thus poverty might prevent a man from seeking justice in the case of his child or wife being raped. At the institutional level, power relations were seen between the law enforcers and the survivors of sexual violence; the law enforcers would reject a case or refer the survivors to community leaders for informal settlements such as compensation, assuming that the survivor had entered into some negotiations with the perpetrator, thus depriving her of justice. The present study suggests that the government should collaborate with key stakeholders in order to reduce violence against women. This is in accordance with WHO recommendations to involve the health sector and create programs to target gender norms that exacerbate the discrimination of women [29]. There are now numerous approaches around the world to modifying gender power relations and tackle these strongly ingrained attitudes. Parents/guardians appear to have a positive attitude towards accepting the introduction of sex and reproductive health education in schools [52]. However, while parents/guardians admitted that it was important for them to talk to their adolescents about sexual and reproductive health issues, traditional cultures still appear to be an inhibiting factor [52]. Engaging men in health programmes has proven positive results [53]. On-going Tanzanian projects, such as “The Champion Project”, focusing on men’s involvement in HIV prevention, and “Men as Partners”, addressing gender roles and reproductive health [54], provide examples on innovative ways to reach men.

### Symbolic dimensions

The symbolic dimension of gender relations emerged as being fundamental in the study context and revolved around the socio-cultural issues that prevented survivors from seeking help and from obtaining appropriate services after experiencing sexual violence. At the societal level, the fear of being blamed for the rape and the resulting stigmatization were the most fundamental barriers to help-seeking of any kind, which explains why reporting rape is often unthinkable. If a woman is raped, it is her reporting of the rape that brings dishonor and shame rather than the violent act itself. These findings are supported by other studies in urban Tanzania where dimensions of social stigma related to sexual violence affect social interactions [33,35,36]. At an individual level, the current study shows that if you are in a marriage, you are forced to remain in that relationship even when you are abused, simply because of the fear of being blamed for disclosing affairs internal to the relationship, but also because marital rape is not recognized by Tanzanian law.

### Emotional dimensions

The emotional dimensions of gender relations often conflicted with both the economic and symbolic dimensions. This finding was very evident at both the intrapersonal and interpersonal levels. For example, young girls were reportedly engaging in sexual relationships with older men through necessity to escape from poverty, but also due to loneliness, especially when parents were absent. In other cases it was considered fashionable for a young school girl to have a boyfriend, or *“Mshefa”* as a way to feel secure, both economically and emotionally. We see emotional dimensions conflicting with symbolic dimensions, especially when a child has been raped; the mother would decide to keep the agony and pain a secret so as to protect her daughter or family’s dignity. Married women who experienced sexual violence would seek solace from elderly people or religious leaders because they feared their partner’s reaction. The lack of anonymity as one distinguished feature of this community and fear of the perpetrator were among barriers to seeking care. The fact that their experience of rape would be known to the whole community was very unpleasant and was linked to avoiding their own internal shame and blame as well as avoidance of receiving direct negative reactions from the community. Similar findings were demonstrated in a study examining barriers to services for rural and urban rape survivors which suggests that a lack of anonymity in rural areas might influence behavior, showing that levels of service utilization and rape reporting differ between rural and urban areas [55].

Our study suggests that the context of rape of women and children is different in rural communities. Given the closed and tight community networks in rural areas, it is unlikely for rape survivors to report or seek care due to fear of community and family reactions [33,35,36,55]. Poverty is characterized by poor access to education and health services, and this might contribute to the normalization of gender roles in which legitimacy for GBV is increased. A high illiteracy rate in this rural community is a major hindrance to justice. Characteristics, such as parental economic circumstances, geography and caste, can set children on an unequal path and trap large groups of people in poverty. These traps can specifically affect access to basic services. This life-long inequality has to be tackled early in order to ensure that today’s children have an equal chance at becoming tomorrow’s successful adults [14]. These findings suggest that interventions in rural areas should focus on educating the community, coordinating service systems, and changing norms and attitudes to be supportive of rape survivors. As recommended by WHO, “macro-level interventions that increase structural supports and resources that decrease gender inequality as well as interventions to reduce gender inequality at the community and individual levels may serve to decrease sexual violence” [56].

### Trustworthiness

Measures to enhance the credibility of the study were taken [35]. Prolonged engagement (over a period of one year) in the field by the first author helped to build trust with the community representatives; this served as a precaution to minimize socially desirable answers during discussions. The first author was responsible for all field work logistics including recruitment of FGDs participants. During data collection, the researchers followed the concept of emergent design by allowing information from the first FGD to guide the subsequent FGDs. Preliminary coding was performed, revised regularly and discussed through peer debriefing sessions with all other co-authors to reach consensus. A flexible guide, emergent design, verbatim transcription and following a structured analytical procedure all aimed to promote the confirmability of the study results. The study is context-based and cannot be directly transferred to other settings. Hence, efforts were made to present rich quotes, provide detailed descriptions of participants and context to facilitate the readers’ judgment of the interpretations and thus the transferability of the results. The qualitative approach involved the researcher as an instrument which has implications in the data collection and analysis. Two of the research team members, MA and PM, are Tanzanians, experienced obstetricians/gynecologists, and native Swahili speakers. The other researchers, PA, ED and PO, reside abroad and have experience in cross-cultural collaboration. PA and ED are obstetricians/gynecologists. PA has worked for several years both clinically and with projects involving GBV both on the African and Asian continents. PO is a nurse midwife, specializing in international qualitative reproductive health research.

### Strengths and limitations of the study

Variation in experience was enhanced by having a diverse and representative sample of informants. The approach of employing FGDs may have given an overly positive view of the community’s perceptions toward rape and child sexual abuse. Our interpretation is that the discussions were free and reflected an increasing awareness about the seriousness of gender- based violence. This was evident when participants expressed their concerns about marital rape, something that was not anticipated as it is against the law as well as culturally taboo to speak about it. Because the topic was sensitive in nature, group discussions are unlikely to allow the expression of viewpoints that counter the dominant public norms, and so we suggest that individual interviews or gender-homogenous FGDs could provide a space in which more pluralistic views can emerge. Having conducted mixed-gender group discussions, it was difficult to analyze the participants’ responses in view of gender differences. Perhaps it would have been easier for women to argue that rape should be recognized within marriage if the groups were structured by gender. Future studies should take this into account to allow gender analysis to take place.

## Conclusions

This study casts light on the many social and gender norms that influence and reinforce sexual violence against women and children. This study also identified prominent socio-cultural and structural barriers that frequently serve as insurmountable obstacles to both seeking and receiving care for sexual violence survivors. Connell’s relational theory of gender enhanced the illumination of the dynamics of gender and sexual violence in the study context. Based on these findings, preventive interventions are needed at different levels (individual, community, institutional, and laws/policies) in order to see long-term promise. At the macro level, legislative reforms, as well as broad investment in strengthening the law enforcement response to GBV, are needed to make the laws work more effectively. Community-based campaigns and mass media “entertainment-education” programs, such as soap operas that address GBV, appear to be promising influences in reducing the tolerance for violence against women. Broad institutional reforms to improve healthcare response to GBV, for example, support provider training, protocols and alliances with referral services, promise to be an effective approach. Multi-sector collaboration is important for most GBV initiatives, especially those that aim to improve women’s lives through social services, economic empowerment and infrastructure improvement. There is a need to create partnerships between government and the non-governmental agencies, as both have a role to play and are unlikely to change the levels of violence by working alone. Addressing the challenges identified here may promote health-seeking behavior and improve the care of survivors of sexual violence, while changes in social norms are needed for the prevention of sexual violence.

## Competing interests

The authors declared no potential conflicts of interest with respect to the authorship and/or publication of this article.

## Authors’ contribution

MA, PM, PA, ED planned the study. MA coordinated the field work logistics. MA and PM collected data. MA, PM, PO and PA conducted the data analysis. MA drafted the manuscript. All authors contributed to the interpretation of the results with their critical comments and assisted in revising the manuscript. All authors read and approved the final manuscript.

## Pre-publication history

The pre-publication history for this paper can be accessed here:

http://www.biomedcentral.com/1472-698X/14/23/prepub
